# Diversity of endophytic fungi from roots of *Panax ginseng* and their saponin yield capacities

**DOI:** 10.1186/2193-1801-2-107

**Published:** 2013-03-13

**Authors:** Hao Wu, Hong-Yan Yang, Xiang-Lin You, Yu-Hua Li

**Affiliations:** College of Life Sciences/Daqing Bio-tech Research Institute, Northeast Forestry University, Harbin, Heilongjiang Province 150040 China

**Keywords:** Diversity, Saponin, Endophytic fungi, *Panax ginseng*, Ginsenoside

## Abstract

Endophytes of medicinal plants have the capacity to synthesis same or similar active substances with their hosts. To investigate the diversity and capacity to produce saponins of endophytic fungi of *Panax ginseng*, thirty-eight strains of were isolated. Polymerase chain reaction (PCR) and sequencing were used to identify the isolates, and saponins concentrations in the cultures were measured. Agar diffusion method was used to test antimicrobial activity. High-performance liquid chromatography (HPLC) was used to analyze ginsenosides produced by representative strains. *Nectria*, *Aspergillus*, *Fusarium*, *Verticillium*, *Engyodontium*, *Plectosphaerella*, *Penicillium*, *Cladosporium*, and *Ascomycete* species were isolated. Overall, 18.4% of the isolates belonged to *Nectria* (*Nectria haematococca*), 13.2% belonged to *Aspergillus*, and 10.5% belonged to *Penicillium*. The highest concentration of triterpenoid saponin was 0.181 mg/ml (Pg27), followed by 0.144 mg/ml (Pg30 and Pg42-1). According to the results of the phylogenetic results, these isolates were species of *Fusarium*, *Aspergillus* and *Verticillium*, respectively. The culture filtrate of Pg30 exhibited its antibacterial activity *Staphylococcus aureus*. Pg 27 and Pg30 could excrete the substances to inhibit the growth of *Rhizoctonia solani*. Pg42-1 showed strong inhibition against *Klebsiella pneumoniae*. From HPLC results, the ginsenoside Rb2 was detected in both Pg27 and Pg30 cultures. The ginsenoside Rc was found in Pg42-1 cultures. In conclusion, thirty-eight endophytic fungal strains were isolated and Pg27 (*Fusarium* sp.) has a potential application value in saponins production.

## Introduction

Ginseng is one of the most famous medicinal plants in the *Araliaceae*, which occupies an important position in traditional Chinese medicine in China. With the excessive and predatory exploitation, wild ginseng resources become scarce. Cultivated ginseng has gradually become the mainstream of the market. To develop the ginseng farming, deforestation reclaimed to new participants is necessary, because the humus in forest area is essential for ginseng cultivation. However, this deforestation has greatly broken the ecological balance and biodiversity in forest area. How to obtain medicinal ingredients from ginseng without damage to the environment has become a very important issue.

Endophytic fungi, which are fungi that colonize a plant without causing visible disease symptoms (Schulza and Boyle 2005), are common in plants (Lin et al. 2010; De Siqueira et al. 2011; Suto et al. 2002), and have been found to be ubiquitous within all examined plants (Sun et al. 2011; Tadych et al. 2012; Li et al. 2012). In addition, endophytic fungi have been isolated from different plant tissues, including flowers, seeds, roots, stems and leaves (Lupo et al. 2001; Bayman et al. 1997). Previous studies have found that some endophytic fungi have roles within the plant in relation to growth (Doty 2011), enhanced stress resistance (Ownley et al. 2010), degradation of pollutants (Sun et al. 2011), and the production of bioactive substances in the host (Guimarães et al. 2008).

In medicinal plants, some endophytic fungi have been found to produce secondary metabolites that have medicinal value. Indeed, since the discovery that the endophytic fungi isolated form *Taxus brevifolia*, *T. celebica*, *T. mairei*, *T. chinensis* var. *mairei*, and *T. wallachiana* produced the anti-cancer drug taxol, many researchers have studied the endophytic fungi of medicinal plants to identify potential sources of novel medicine (Lin et al. 2007; De Siqueira et al. 2011; Kumaran et al. 2010). Saponin is the main medicinal product of *Panax ginseng* and has multiple therapeutic values, including anti-tumor and anti-aging properties and blood vessels softening. Studying the saponin yield capacity of *Panax ginseng* endophytes could provide new sources for producing saponins and protect wild ginseng resources indirectly. Antimicrobial activity of endophytes is also one research direction. Endophytes and their metabolites are generally not harmful to their host. Therefore, endophytes which are resistant to pathogens may become the natural sources for pesticides (Yang et al. 2006).

Researches on ginseng endophytes mainly focused on the diversity and the biological activity of metabolites. Xu et al. isolated *Paecilomyces* sp. from the ginseng and studied its antifungal and antitumor properties. The results showed that the extracts derived from *Paecilomyce* sp. and ginseng samples contained the same compound falcarinol, an atural pesticide and anti-cancer agent (Xu et al. 2009). Park et al. isolated 38 fungal isolates from three cultivars of *Panax ginseng* in Korea. They were classified into *Phoma radicina*, *Fusarium oxysporum*, *Setophoma terrestris* and *Ascomycota* in 3 cultivars (Park et al. sp. 2-RNK. The most dominant fungal endophyte was *P. radicina*^2012^). In the present study, to select the endophytes with the capacity of producing saponins, we investigated the diversity of the endophytic fungi in the roots of *Panax ginseng* cultivated in the forest of Northeast China. The saponin concentrations of typical strains were measured. The antimicrobial activity of representative strains was tested and ginsenosides produced by typical strains were analyzed.

## Materials and methods

### Sampling and isolation

*Panax ginseng* (PG) specimens were respectively sampled from Fu-yuan City and Ji-an City (Jinlin, China) and had been grown for 15 years in the forest. The PG samples were immediately put into sterile plastic bags and stored at 4°C. The endophytes were isolated within 48 hours.

Before disinfection, the plant samples were thoroughly washed under running tap water for 10 h. The PG root samples were surface-disinfected with 70% (v/v) ethanol for 1 min, 5% NaOCl 10 min, 70% (v/v) ethanol for 1 min and burning for 30 sec. The samples were subsequently rinsed with sterile water, and the outer tissue was removed with a sterile scalpel. Small pieces (0.5×0.5 cm) of PG were placed in Petri dishes containing malt extract agar (MEA, Difco, USA), Czapeck agar (CZA, Difco, USA), or potato dextrose agar (PDA, Difco, USA), and incubated at 28°C for five days. Following the incubation, single colonies of distinctive morphotypes were separated on the basis of their morphological characteristics and appearance. The colonies were subsequently re-isolated by plating on PDA and incubated at 28°C for 24–48 h to obtain pure cultures. All of the isolates were vacuum freeze-dried and deposited in the College of Life Sciences, Northeast Forestry University.

### DNA extraction and PCR amplification of the 28S rRNA gene

The genomic DNA was extracted using the EZNA Fungal DNA Mini Kit (OMEGA, USA) according to the manufacturer’ instructions. The 50 μl PCR mixtures contained 15 ng of template DNA, 1× PCR buffer (Mg^2+^ free), 0.16 mM of each dNTP, 1.5 mM MgCl_2_, 0.45 μM of each primer, and 1 U of Takara *rTaq* DNA polymerase (Takara, Japan). The primers for the amplification of the D1/D2 region of the fungal 28S rRNA gene were NL1 (5’-GCATATCAATAAGCGGAGGAAAAG-3’) and NL4 (5’-GGTCCGTGTTTCAAGACGG-3’)(Redecke 2000). The thermocycler program consisted of initial an DNA denaturation at 95°C for 5 min followed by 30 cycles of denaturation at 95°C for 1 min, annealing at 52°C for 45 s, and elongation at 72°C for 1 min 30 s, and ending with a final elongation step at 72°C for 6 min (Yang et al. 2007).

The PCR amplification products were separated by electrophoresis through 1% (W/V) agarose gels and stained with ethidium bromide for visual examination. The PCR products were purified using the Agarose Gel DNA Extraction Kit (Takara, Japan) and sequenced at Sangon Bioteck (Shanghai, China).

### Phylogenetic analysis and nucleotide sequence accession numbers

The sequences generated in this study were compared with those in GenBank ( http://blast.ncbi.nlm.nih.gov/Blast.cgi), and the sequences with a similarity ≥99% to the partial 28S rDNA regions (a. 600 bp) were considered to belong to identical genera. A neighbor-joining tree (Thompson et al. 1997) was constructed using MEGA 5.0 software (Tamura et al. 2011). The number of bootstrap replications was 1000. The sequences were deposited in GenBank under the accession numbers shown in Table 1.

**Table 1:** **Similarity between the isolates and closest species in GenBank**

Strain ID	Accession No.	Closest species (Accession No.)	Similarity (%)
Pg31	JQ807916	*Verticillium* sp. (AY312607)	99.5
		*Engyodontium album* (HM214541)	99.3
		*Engyodontium album* (DQ872372)	97.9
Pg50-1	JQ807940	*Fusarium* sp. (AB294824)	100
Pg14	JQ807941	*Fusarium* sp. (AB294823)	100
		*Fusarium solani* (AB363765)	100
Pg33-2	JQ807905	*Penicillium guttulosum* (HQ646592)	100
Pg33	JQ807906	*Penicillium menonorum* (HQ646591)	100
		*Penicillium menonorum*(HQ646590)	99.8
Pg42-1	JQ807917	*Verticillium* sp. (HM057107)	100
		*Verticillium psalliotae* (AF500907)	100
		*Verticillium psalliotae* (AB378520)	99.6
Pg44	JQ807958	Uncultured *Ascomycota* (HQ432963)	100
Pg4-2	JQ807959	*Plectosphaerella cucumerina* (JF780520)	100
Pg64	JQ807960	*Plectosphaerella cucumerina* (HQ239034)	100
Pg4-1	JQ807961		
Pg42	JQ807911	*Aspergillus fumigatus* (JQ268555)	100
Pg41-2	JQ807912	*Aspergillus fumigatus* (JN938928)	100
		*Aspergillus fumigatus* (AB354184)	100
Pg34	JQ807970	Fungal sp. (GU552503)	99.6
		*Fusarium oxysporum* (FJ614650)	99.6
		*Fusarium* sp. (AB373725)	99.6
Pg50	JQ807951	*Penicillium simplicissimum* (HM469430)	99.5
Pg50-1	JQ807940	*Penicillium* sp. (HM469409)	99.5
		*Penicillium brasilianum* (HM469396)	99.5
Pg61	JQ807988	Uncultured *Ascomycota* (HQ433122)	95.6
		Uncultured *Ascomycete* (HQ432972)	95.5
		Uncultured *Ascomycete* (EU489938)	93.4
Pg32	JQ807987	*Penicillium guttulosum* (HQ646592)	92.4
		*Penicillium menonorum* (HQ646591)	92.4
		*Penicillium menonorum* (HQ646590)	92.2
Pg10	JQ807978	Uncultured *Ascomycota* (HQ433122)	99.6
Pg5	JQ807980	*Paraphoma chrysanthemicola* (GQ387582)	98.9
Pg63	JQ807979	*Paraphoma chrysanthemicola* (GQ387583)	98.9
Pg12-1	JQ807937	*Fusarium* sp. (AB294826)	100
		*Nectria haematococca* (DQ119558)	99.8
		*Nectria haematococca* (HM042416)	99.8
Pg36	JQ807948	Fungal sp. (GU552494)	100
		*Neonectria radicicola* (HM364304)	99.6
		*Neonectria radicicola* (U17415)	99.3
Pg34	JQ807970	*Cladosporium* sp. (FJ790290)	100
		*Cladosporium cladosporioides* (AY213695)	100
		*Passalora fulva* (AB100653)	100
Pg40	JQ807971	*Cladosporium cladosporioides* (JN651416)	100
		*Cladosporium silenes* (JF770463)	100
		Uncultured *Cladosporium* (JF449832)	100
Pg30-1	JQ807913	*Aspergillus sydowii* (GU004536)	100
Pg30	JQ807914	*Aspergillus protuberus* (GQ132189)	100
Pg5-1	JQ807915	*Aspergillus sydowii* (EF652473)	100
Pg27	JQ807957	*Fusarium subglutinans* (HQ876767)	100
		*Fusarium proliferatum* (HQ332533)	100
		*Fusarium* sp. (EU193176)	100
Pg16-1	JQ807977	Uncultured *Pleosporales* (JF691161)	100
		Uncultured *Epicoccum* (JF449817)	100
		Uncultured *Epicoccum* (JF449816)	100
Pg6	JQ807936	*Nectria haematococca* (HM042416)	100
Pg28	JQ807934	*Nectria haematococca* (AB373719)	100
Pg79	JQ807933	*Nectria haematococca* (AB513852)	100
Pg42-2	JQ807932		
Pg41-1	JQ807931		
Pg41	JQ807930		
Pg45	JQ807919	Uncultured soil *fungus* (EU691410)	100
Pg60	JQ807920	Uncultured soil *fungus* (EU691436)	100
Pg47	JQ807921	Uncultured soil *fungus* (EF639724)	99.5

### Determination of triterpenoid saponins

Each isolate was cultured in 100 ml PDA liquid medium (250 ml flask), and stirred at 150rpm at 28°C for two weeks. After ultrasonication, the supernatant was separated from the cell debris by centrifugation at 4,000 × *g* for 20 min. A 20 ml aliquot of the supernatant was poured into a 50 ml centrifuge tube (Corning, USA), and 20 ml ethyl acetate was added into the same tube. After mixing, ultrasonication and incubation for 5 min, 5 ml of the supernatant was evaporated to dryness under a vacuum at 50°C. The residue was dissolved in 2 ml methanol. The methanol solutions were centrifuged at 4,000 × *g* for 10 min, and the supernatants were used for the subsequent analysis of the total saponins and ginsenoside.

The measurement of the total extracted saponins was based on a color reaction of the acid-hydrolysis products of the saponins (i.e. sapogenins) with vanillin. In total, 5 ml of the supernatant was added to a test tube and evaporated at 60°C in a water bath. The residue was dissolved in 0.2 ml 5% vanillin, mixed with 0.8 ml perchloric acid, incubated at 60°C in a water bath for 15 min and quickly cooled in ice water. The concentration of saponins (mg/ml) in the reaction sample was detected using a spectrophotometer at 560 nm against a calibration curve established with an oleanolic acid standard (National Institutes for Food and Drug Control, Bei-jing, China) (Liu et al. 2011).

### Antimicrobial activity of the representative strains

The 14-day culture filtrates were assessed for antimicrobial activity by the agar diffusion method (Hormazabal and Piontelli 2009) against the test microorganism strains showed as Table 3. Three 6-mm wells were made in each disk. Culture filtrates (0.2 ml) was added in each well. Except for *Fusarium sporotrichioides* (isolated in our lab), the other strains were purchased from Agricultural Culture Collection of China (ACCC). As a reference, the Streptomycin Sulfate (5 mg/well), the Amoxicillin (5 mg/well) and the Itraconazole Hydrochloride (4.4 mg/well) were used as antibacterial standards. The activity of the extracts was estimated from growth inhibition (in mm).

### Ginsenosides analyses

A 100 ml ethyl acetate was added the 100 ml liquid culture. After 30 min agitation at 160 rpm and ultrasonication at 50°C, the supernatant was separated from the cell debris by centrifugation at 4,000 × *g* for 30 min. After evaporation, the pellet was dissolved with a 5 ml methanol, then filtrated with SepPak C-18 Cartridge (Waters, USA). Standards were purchased from National Institutes for Food and Drug Control (Bei-jing, China). Acetonitrile (DIKMA, USA) and water were HPLC grade. HPLC analysis were performed using Separations Module (Model e2695, Waters, USA), photodiode Array Detector (Model 2998, Waters). Sample volume was 10 μl. The wavelength of the detector is 203 nm. Ginsenoside was analyzed using a XTerra® MS column C-18, 5 μm, 4.6 mm × 2.5 mm. The mobile phase consisted of a mixture, acetonitrile :water (0-40 min, 18:82–18:82, v/v; 40-50 min, 18:80–22:78 v/v; 50-70 min, 22:78–28:72 v/v; 70-100 min, 28:72–38: 62 v/v; 100-110 min, 38:62–18:82 v/v;). The flow was of 1.0 ml min^-1^ and the sensitivity was 0.001 AUFS. The HPLC system was operated at room temperature (25 ± 1°C).

## Results

### Similarity of the sequences

Thirty-eight strains were identified on the basis of their morphological characteristics. The sequences were compared with those in the GenBank database, and the results are shown in Table 1.

### Phylogenetic analysis

**Figure 1:**
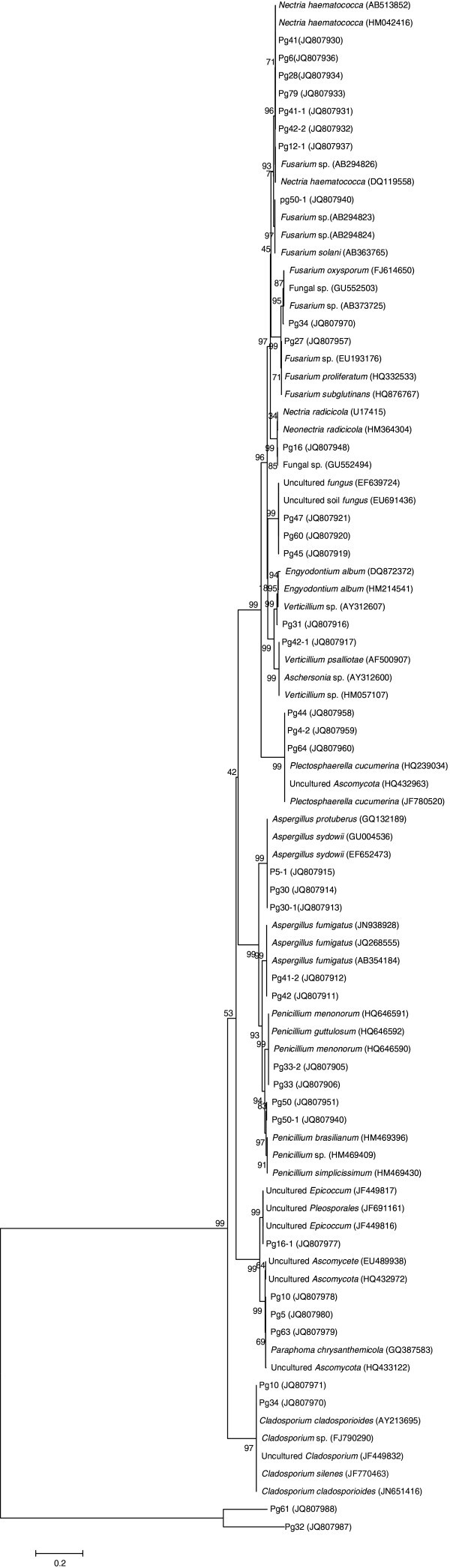
**Phylogenetic tree based on 28S rDNA sequences using Neighbor-Joining method.** Scale bar indicates 20% estimated sequence divergence.

The phylogenetic tree built from the 28S rDNA sequences is shown in Figure 1. Nine fungal genera were identified: *Nectria*, *Aspergillus*, *Fusarium*, *Verticillium*, *Engyodontium*, *Plectosphaerella*, *Penicillium*, *Cladosporium*, and *Ascomycete*. The most representative genera were *Nectria*, *Aspergillus*, and *Penicillum*: 18.4% belonged to *Nectria*, (*Nectria haematococca*), 13.2% belonged to *Aspergillus*, and 10.5% belonged to *Penicillium*.

### Analysis of triterpenoid saponins

The concentration of triterpenoid saponins of typical isolates are showed in Table 2 The highest concentration of saponins was 0.181 mg/ml in Pg27, which was significantly higher than Pg30 and Pg42-1 (0.144 mg/ml) (*P*<0.05). According to the results of phylogenetic results, Pg27 was identified as a *Fusarium* sp., Pg30 was identified as an *Aspergillus* sp., and Pg42-1 was identified as a *Verticillium* sp. .The saponin concentrations among the strains of the same genus were different significantly (*P*<0.05), for example Pg14 (0.023 mg/ml), Pg34 (0.133 mg/ml) and Pg12-1 (0.042 mg/ml); these isolates were also identified as *Fusarium* spp*.*

**Table 2:** **Analysis of*Panax ginseng*triterpenoid saponins in typical isolates**

Isolate ID	Mean±Stdev (mg/ml)	Significance ( ***P*** <0.05)	Closest species (Accession No.)
Pg27	0.181±0.006	a	*Fusarium subglutinans* (HQ876767)
Pg30	0.144±0.002	b	*Aspergillus sydowii* (GU004536)
Pg42-1	0.144±0.009	b	*Verticillium* sp. (HM057107)
Pg33-2	0.136±0.004	c	*Penicillium guttulosum* (HQ646592)
Pg34	0.133±0.002	c	*Fusarium oxysporum* (FJ614650)
Pg41-2	0.130±0.002	c	*Aspergillus fumigatus* (JQ268555)
Pg42-2	0.115±0.003	d	*Nectria haematococca* (HM042416)
Pg50-1	0.109±0.004	d	*Penicillium simplicissimum* (HM469430)
Pg61	0.079±0.004	e	Uncultured *Ascomycota* (HQ433122)
Pg32	0.072±0.003	f	*Penicillium guttulosum* (HQ646592)
pg10	0.071±0.005	f	Uncultured *Ascomycota* (HQ433122)
pg41	0.063±0.003	g	*Nectria haematococca* (HM042416)
Pg40	0.059±0.002	gh	*Cladosporium cladosporioides* (JN651416)
Pg45	0.050±0.003	hi	Uncultured soil fungus (EU691410)
Pg44	0.052±0.002	ij	*Plectosphaerella cucumerina* (JF780520)
Pg36	0.048±0.003	jk	Fungal sp. (GU552494)
Pg12-1	0.042±0.003	k	*Fusarium* sp. (AB294826)
Pg31	0.035±0.003	l	*Verticillium* sp. (AY312607)
Pg14	0.023±0.002	m	*Fusarium* sp. (AB294824)

### Antimicrobial activity

To test the Pg27, Pg30 and Pg42-1 potential use, the antimicrobial activity was analyzed. From Table 3, the culture filtrate of Pg30 exhibited its antibacterial activity against Gram-positive bacteria *Staphylococcus aureus* ACCC10499. Pg 27 and Pg30 could excrete the substances to inhibit the growth of *Rhizoctonia solani* ACCC36233, which was a pathogenic fungi of *Panax notoginseng*. The culture filtrate of Pg42-1 showed strong inhibition against *Klebsiella pneumoniae* ACCC10498. This result indicated that Pg42-1 might be a potential medical source.

**Table 3:** **Antimicrobial activity of representative endophytic fungi strains**

Test strains	Representative strains		
	Pg27	Pg30	Pg42-1
*Staphylococcus aureus* ACCC10499	-	++	-
*Bacillus subtitis* ACCC10243	-	-	-
*Klebsiella pneumoniae* ACCC10498	-	-	+++
*Pseudomonas aeruginosa* ACCC10500	-	-	-
*Phytophthora cactorum* ACCC36421	-	-	-
*Rhizoctonia solani* ACCC36233	++	++	-
*Aspergillus niger* ACCC30005	-	-	-
*Fusarium sporotrichioides*	-	-	-

Culture filtrate (0.2 ml) was added in each well (6 mm); (−) no inhibiton, (+) inhibition zone, +++ width of growth inhibition zone > 10 mm, ++ 5–10 mm, + 1-5 mm;

### Ginsenosides analyses

**Figure 2:**
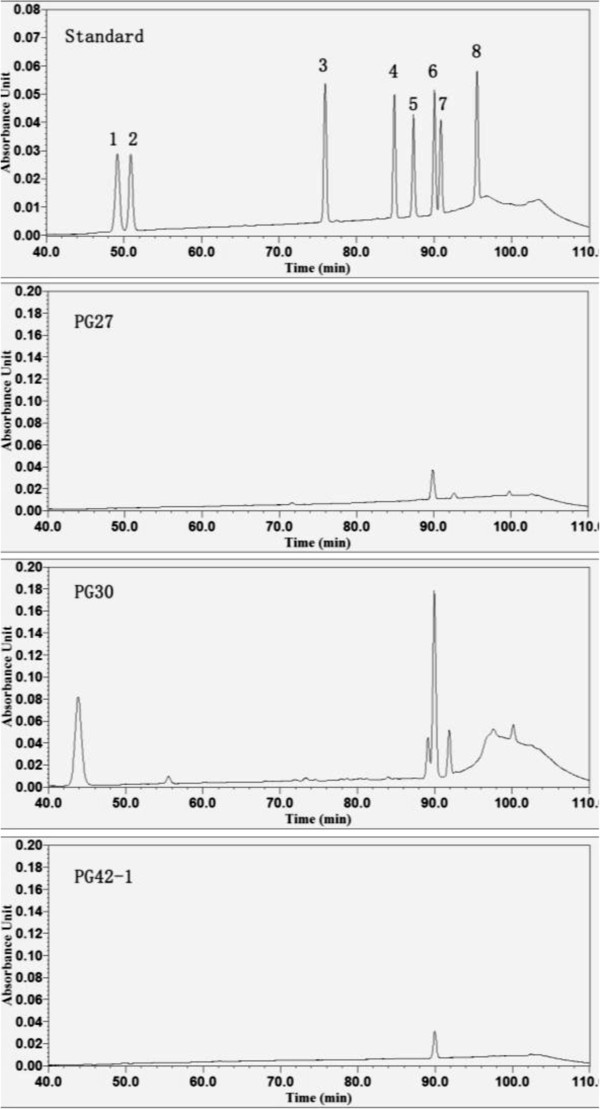
**HPLC spectrums of culture filtrates from the representative strains.** 1, Rg1; 2, Re; 3, Rf; 4, Rb1; 5, Rc; 6, Rb2; 7, Rb3; 8, Rd.

Accoridng the result of total saponins, Pg27, Pg30 and Pg42-1 produced higher concentrations of saponins. To further analyze the composition of saponins, the standards of eight ginsenosides were injected into HPLC. The spectrums showed as Figure 2. Rb2 was detected in both Pg27 and Pg30 cultures. The concentration of Pg30 was especially high. Rc was found in Pg42-1 cultures.

## Discussion

Thirty-eight endophytic fungi were isolated, and they were classified into nine genera according to the morphological types and 28S rDNA sequencing results. Three isolates (Pg45, Pg47, and Pg60) were not identified because of lack of comparative sequences: their sequences were significantly similar to unknown fungal sequences in the GenBank database. *Nectria*, *Aspergillus* and *Penicillium* were the predominant genera. The host materials were all healthy in this study. Park et al. ( 2012) reported that *Phoma radicina*, *Fusarium oxysporum*, *Setophoma terrestris* and *Ascomycota* were the predominant endophytic fungi in Korean Ginseng, and Xing et al. ( 2010) reported that *Cladosporium* sp. was the dominant species in the root of *Panax quinquefolium*. These previous results were different from this present study, indicating the specificity of the endophytic fungi from different areas and plants.

*Nectria* was reported as endophytic fungi in European beech ( Danti et al. 2002) and red alder ( Dorworth et al. 1996) as the endophytic fungi. *Nectria* has also been associated with the canker diseases of tree species. However, the *Nectria* isolates in this study didn’t show their pathogenicity to the host plant. Therefore, the pathogenicity of *Nectria* had their specificity. *Aspergillus* species could be sources of new medicines. For example, Kusari and Zhao had reported that an *Aspergillus* sp. was a source of anti-cancer medicines ( Kusari et al. 2009; Zhao et al. 2009). Therefore, further research on the *Aspergillus* sp. isolated in this study may be interesting. *Penicillium* is the source of penicillin, and recent results showed that endophytic *Penicillium* sp. had the capacity to secrete anti-tumor substances ( Aly et al. 2010) or hypocrellin ( Meng et al. 2011). We propose that the endophytic fungi isolated in this study from a medical plant are potential sources of medicines.

The growth-promotion factors and metabolites produced by endophytic fungi have been widely applied in medicine and agriculture. The most famous substance is taxol, a mitotic inhibitor used in cancer chemotherapy, which was originally produced by the yew tree and can be produced by endophytic fungi of yew trees ( Rivera-Orduña et al. 2011). Similarly, a filterd liquid culture of endophytic fungi was analyzed to identify endophytes that could produce triterpenoid saponins. Overall, 19 of the isolated fungi showed a color reaction, which indicated that they could produce triterpenoid saponins. Among them, Pg27 (*Fusarium* sp.), Pg30 (*Aspergillus* sp.) and Pg42-1(*Verticillium* sp.) exhibited higher concentrations of total saponins. These three isolates could be good candidates for further studies on their capacity to produce possible medical substances. *Fusarium* spp. were the main endophytes isolated from winter wheat ( Sieber et al. 1988) and soybean ( Pimentel et al. 2006). Many studies have shown that *Fusarium* spp. isolated from banana and tomato have the capacity to inhibit nematodes ( Vu et al. 2004; Pocasangre et al. 1999; Hallmann and Sikora 1996)**.** Phongpaichit et al. studied the antimicrobial activity of the endophytic fungi isolated from *Garcinia* species were different (Phongpaichit et al. species, with the results showing that the antimicrobial activities from different *Garcinia*.

In the present study, several *Fusarium* spp. were isolated from the PG roots, and some could produce bioactive saponins. The concentrations of saponins from the different isolates were significantly different (*P*<0.5), suggesting that their capacities to produce saponins were different. Further characterization of the bioactive compounds produced by fungi with high saponin-producing capacities could provide the possibility to obtain medical substances. HPLC results indicated that these three strains, as their host plants, had the capacity to produce some ginsenosides of *Panax ginseng*. Further research to improve their capacity of producing some ginsenosides is necessary. This work is under way.
